# Active Vitamin D Level Is Independently Associated with the Presence and Severity of Coronary Artery Disease in Patients with Chronic Kidney Disease

**DOI:** 10.3390/medicina62010124

**Published:** 2026-01-07

**Authors:** Il Young Kim

**Affiliations:** 1 Department of Internal Medicine, Pusan National University School of Medicine, Yangsan 50612, Republic of Korea; iykim@pusan.ac.kr; 2 Research Institute for Convergence of Biomedical Science and Technology, Pusan National University Yangsan Hospital, Yangsan 50612, Republic of Korea

**Keywords:** coronary artery disease, chronic kidney disease, vitamin D

## Abstract

*Background and Objectives:* Chronic kidney disease (CKD) increases the risk of coronary artery disease (CAD), and vitamin D deficiency—particularly reduced levels of 1,25-dihydroxyvitamin D [1,25(OH)_2_D], the biologically active form of vitamin D that declines early in CKD due to impaired renal conversion—may be a contributing factor. This study aimed to assess the relationship between 1,25(OH)_2_D levels and the presence and severity of CAD in CKD patients. *Materials and Methods:* We retrospectively analyzed 398 non-dialysis CKD patients (eGFR < 60 mL/min/1.73 m^2^) who underwent elective coronary angiography. Serum 1,25(OH)_2_D and 25(OH)D levels were measured, and CAD severity was assessed using the Gensini score. *Results:* Lower 1,25(OH)_2_D levels were independently associated with both the presence and se-verity of CAD. Logistic regression revealed that each 1 pg/mL increase in 1,25(OH)_2_D was linked to an 11% reduction in odds of significant CAD (OR: 0.89; 95% CI: 0.86–0.93; *p* < 0.001). In contrast, 25(OH)D was not significantly related to CAD. Linear regression showed an inverse correlation between 1,25(OH)_2_D and Gensini scores (β = −0.329, *p* < 0.001), indicating reduced disease severity with higher vitamin D levels. Subgroup analyses confirmed consistent associations across age, sex, diabetes, hypertension, and LDL-cholesterol categories. ROC analysis demonstrated that 1,25(OH)_2_D alone had good predictive ability for CAD (AUC = 0.818), which improved to 0.925 when combined with traditional risk factors. The optimal cutoff for 1,25(OH)_2_D was ≤16.6 pg/mL, yielding 73.3% sensitivity and 83.5% specificity. *Conclusions*: Serum 1,25(OH)_2_D is an independent predictor of both the presence and extent of CAD in CKD patients and may serve as a valuable non-traditional biomarker for cardiovascular risk assessment.

## 1. Introduction

Chronic kidney disease (CKD) represents a major health concern, leading to adverse patient outcomes and substantial healthcare expenditures [1,2]. In individuals with CKD, cardiovascular disease (CVD) stands as the predominant cause of mortality, with even mild declines in renal function being strongly correlated with an increased risk of cardiovascular complications and death [3]. This trend is also evident in the development of Coronary Artery Disease (CAD) in CKD patients [4]. Despite intensive efforts to control traditional CAD risk factors, such as high blood glucose levels and hypertension, CAD continues to occur more frequently and with higher mortality rates in CKD patients compared to those without CKD [4]. This suggests that additional, non-traditional risk factors unique to CKD may contribute to CAD development. Therefore, identifying CKD-specific risk factors, in addition to established traditional risk factors, is essential for improving CAD outcomes in this population.

Vitamin D deficiency is frequently observed in individuals with CKD and results from both reduced levels of 25-hydroxyvitamin D [25(OH)D] and impaired function of 1α-hydroxylase, the enzyme responsible for converting 25(OH)D into its active form, 1,25-dihydroxyvitamin D [1,25(OH)_2_D] [5]. Previous research has highlighted the diverse physiological roles of vitamin D beyond its well-established involvement in bone and mineral metabolism, with deficiency in this vitamin being linked to increased mortality and unfavorable cardiovascular outcomes in both the general population and CKD patients [6,7]. Among CVDs, an elevated incidence and mortality of CAD have been associated with vitamin D deficiency in individuals undergoing coronary angiography (CAG) [6]. Moreover, accumulating evidence suggests a correlation between vitamin D deficiency and both the presence and severity of CAD in patients undergoing CAG [8,9,10,11,12,13,14]. However, the precise relationship between vitamin D status and CAD in CKD patients remains unclear.

Most previous studies investigating the relationship between vitamin D status and CAD have focused on serum 25(OH)D levels. However, 25(OH)D primarily reflects vitamin D stores and does not necessarily indicate biological activity, particularly in patients with CKD. In CKD, impaired renal 1α-hydroxylase activity leads to reduced conversion of 25(OH)D to its active form, 1,25(OH)_2_D, even when circulating 25(OH)D levels are relatively preserved. As a result, 1,25(OH)_2_D may serve as a more physiologically relevant marker of vitamin D status in this population. Despite this, limited data are available regarding the association between 1,25(OH)_2_D levels and the presence or severity of coronary artery disease in patients with CKD. Therefore, the present study aimed to investigate the relationship between serum 1,25(OH)_2_D concentrations and both the presence and severity of coronary artery disease in individuals with CKD.

Given the high prevalence of both CAD and vitamin D deficiency in CKD patients, we recognized the need to further explore this potential connection. Therefore, this study aims to investigate the association between vitamin D levels and the presence and severity of CAD in individuals with CKD.

## 2. Materials and Methods

### 2.1. Study Cohort

This investigation retrospectively analyzed the medical records of 398 individuals aged 18 years or older who underwent CAG between 2010 and 2023, with available serum 1,25(OH)_2_D and 25(OH)D measurements. Only first-time elective CAG cases were included, while those who had undergone emergency CAG were excluded. Participants with a prior history of coronary revascularization, congenital cardiac anomalies, or cardiomyopathy were not considered. The estimated glomerular filtration rate (eGFR) was determined utilizing the Chronic Kidney Disease Epidemiology Collaboration (CKD-EPI) equation. All included patients had CKD, with an eGFR below 60 mL/min/1.73 m^2^, and none were receiving dialysis. Individuals who had taken vitamin D supplements, calcimimetics, or other agents that could alter endogenous vitamin D level were omitted from the study. The research protocol adhered to the principles of the Declaration of Helsinki and relevant ethical guidelines, with approval granted by the Institutional Review Board (IRB) of Pusan National University Yangsan Hospital (IRB No. 55-2025-105, approval date: 29 August 2025). Given the retrospective nature of the study, which utilized anonymized clinical data, the IRB waived the requirement for informed consent.

### 2.2. Collected Data

Demographic and clinical details, including age, sex, smoking status, diabetes, and hypertension, were recorded. Body mass index (BMI) was computed using weight and height and expressed as kg/m^2^. Hypertension was characterized by either antihypertensive medication use or blood pressure exceeding 140/90 mmHg. Diabetes was defined by the use of antidiabetic medication, a fasting blood glucose level of at least 126 mg/dL, or a hemoglobin A1c reading of 6.5% or higher. Laboratory parameters such as albumin, uric acid, calcium, phosphate, low-density lipoprotein (LDL) cholesterol, hemoglobin, high-sensitivity C-reactive protein (hsCRP), intact parathyroid hormone (PTH), 25(OH)D, and 1,25(OH)_2_D levels were measured concurrently. The urinary albumin concentration was determined using the urinary albumin-to-creatinine ratio (mg/g Cr). Serum 25(OH)D concentrations were quantified via a chemiluminescence immunoassay (DiaSorin, Saluggia, Italy; reference range: 4.8–52.8 ng/mL), while 1,25(OH)_2_D levels were assessed with a radioimmunoassay (DIAsource ImmunoAssays, Louvain-la-Neuve, Belgium; reference range: 19.6–54.3 pg/mL).

### 2.3. Coronary Angiographic Assessment

All participants underwent elective CAG, meeting the inclusion criteria. Three experienced cardiologists independently reviewed the angiographic findings. CAD was classified as significant if at least one major coronary artery exhibited lumen narrowing of 50% or more. The extent of CAD severity was quantified using the Gensini score. Scores were assigned based on the degree of luminal obstruction: 1 point for 1–25% stenosis, 2 for 26–50%, 4 for 51–75%, 8 for 76–90%, 16 for 91–99%, and 32 for total occlusion. Each score was further adjusted using weighting factors based on lesion location, such as 5 for the left main artery, 2.5 for the proximal left anterior descending and left circumflex arteries, and 1 for the proximal right coronary artery. The final Gensini score represented the cumulative sum of all lesion scores.

### 2.4. Statistical Approach

Continuous variables were expressed as medians with interquartile ranges and compared using Kruskal–Wallis tests. Categorical variables were presented as frequencies and percentages and analyzed using the chi-square test. The relationship between different variables and significant CAD was assessed through univariable and multivariable logistic regression models to estimate odds ratios (ORs) and their respective 95% confidence intervals (CIs). The correlation between clinical parameters and Gensini score was evaluated using Pearson’s correlation for univariable analysis and a multivariable linear regression model. Variables found to be significant in univariable analyses were included, along with clinically relevant covariates, for multivariable adjustments. Receiver operating characteristic (ROC) curve analysis was employed to determine the area under the curve (AUC), while the Youden index was utilized to establish the optimal threshold for serum 1,25(OH)_2_D levels in identifying significant CAD. Logistic regression was used to assess the AUC for combined factors, with predictive probabilities calculated accordingly. ROC curves were generated based on these probabilities, and AUC comparisons were conducted using the methodology proposed by DeLong et al. A two-tailed *p*-value below 0.05 was considered statistically significant. All statistical computations were performed using SPSS version 27.0 (SPSS, Inc., Chicago, IL, USA) and MedCalc Statistical Software version 22.023 (MedCalc Software, Ostend, Belgium).

## 3. Results

### 3.1. Study Population Characteristics

Table 1 illustrates the demographic and clinical features of participants categorized by serum 1,25(OH)_2_D concentrations. Individuals with higher serum 1,25(OH)_2_D levels were generally older. There were no notable differences among the groups regarding gender distribution or smoking habits. Those in the upper tertiles exhibited a lower prevalence of diabetes and hypertension. Moreover, eGFR progressively increased with rising serum 1,25(OH)_2_D levels. Participants with elevated serum 1,25(OH)_2_D had higher hemoglobin concentrations but demonstrated lower urinary albumin, phosphate (*p* < 0.001), LDL-cholesterol, hsCRP, and intact PTH levels. Regarding CAD severity, individuals with greater serum 1,25(OH)_2_D concentrations exhibited a reduced likelihood of significant CAD (prevalence: 95.5% vs. 69.9% vs. 43.6%, *p* < 0.001) and had lower Gensini scores (median values: 74 vs. 32 vs. 10, *p* < 0.001).

### 3.2. Relationship Between Vitamin D Levels and CAD

Table 2 outlines factors correlated with the presence of significant CAD. In the univariate logistic regression model, serum 1,25(OH)_2_D levels (per 1 pg/mL, OR: 0.89, 95% CI: 0.87–0.91, *p* < 0.001) showed a strong inverse relationship with significant CAD, whereas 25(OH)D levels did not exhibit a similar association. Additional factors linked to significant CAD included age, smoking status, diabetes, hypertension, eGFR, urinary albumin, phosphate, LDL-cholesterol, hsCRP, and intact PTH. When adjusting for multiple variables, serum 1,25(OH)_2_D levels (per 1 pg/mL, OR: 0.89, 95% CI: 0.86–0.93, *p* < 0.001) remained significantly associated with the presence of CAD. Other independent factors included age (per year increase, OR: 1.08, 95% CI: 1.02–1.09), smoking (current vs. non-smoker, OR: 7.36, 95% CI: 2.41–22.46, *p* < 0.001), diabetes (yes vs. no, OR: 3.85, 95% CI: 1.91–7.77, *p* < 0.001), hypertension (yes vs. no, OR: 3.52, 95% CI: 1.79–6.95), and LDL-cholesterol (per 1 mg/dL, OR: 1.02, 95% CI: 1.10–1.03).

**Figure 1:**
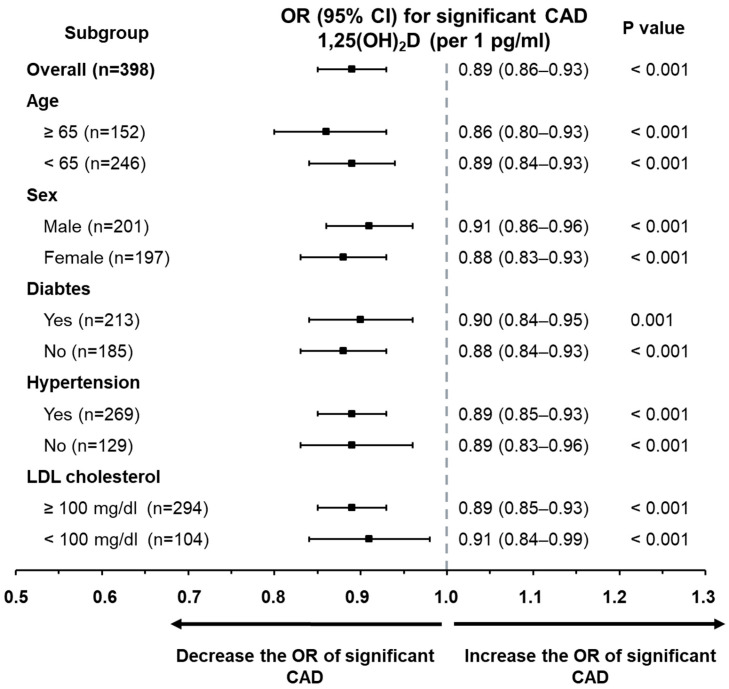
Subgroup analysis displaying the odds ratios of 1,25(OH)_2_D for predicting clinically significant CAD. Through multivariate logistic regression, 1,25(OH)_2_D emerged as an independent factor associated with significant CAD across various predefined subpopulations. These included distinctions based on age (≥65 vs. <65 years), gender (male vs. female), diabetic status, presence of hypertension, and levels of low-density lipoprotein cholesterol (≥100 mg/dL vs. <100 mg/dL).

Further subgroup analyses (Figure 1) confirmed that serum 1,25(OH)_2_D concentrations maintained an independent inverse association with significant CAD across different stratifications, including age (≥65 vs. <65 years), sex, presence of diabetes or hypertension, and LDL-cholesterol levels (≥100 mg/dL vs. <100 mg/dL).

Table 3 demonstrates the factors related to Gensini scores among participants. In the univariate linear regression analysis, serum 1,25(OH)_2_D levels were significantly inversely correlated with Gensini scores (r = −0.539, *p* < 0.001), whereas 25(OH)D levels did not show a similar correlation. Other variables related to Gensini scores included age, gender, smoking, diabetes, hypertension, eGFR, urinary albumin, phosphate, LDL-cholesterol, hemoglobin, hsCRP, and intact PTH. In the multivariate linear regression analysis, serum 1,25(OH)_2_D concentrations (β = −0.329, *p* < 0.001) remained strongly associated with Gensini score. Additional significant factors included age (β = 0.203, *p* < 0.001), smoking status (β = 0.126, *p* < 0.001), diabetes (β = 0.187, *p* < 0.001), hypertension (β = 0.149, *p* < 0.001), and LDL-cholesterol (β = 0.162, *p* < 0.001).

### 3.3. Diagnostic Performance of 1,25(OH)_2_D for Significant CAD

**Figure 2:**
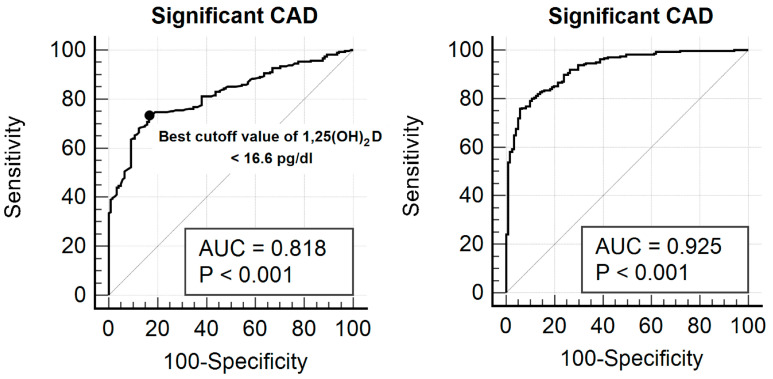
ROC curve illustrating the diagnostic performance of serum 1,25(OH)_2_D concentration for identifying significant CAD in the study cohort. The calculated AUC was 0.818 (95% confidence interval: 0.777–0.855). The best cut-off value for 1,25(OH)_2_D was determined to be ≤16.6 pg/mL, yielding a sensitivity of 73.3% and specificity of 83.5%. When additional independent risk variables—such as age, tobacco use, diabetes, hypertension, and LDL cholesterol—were included in the predictive model, the AUC rose to 0.925 (95% CI: 0.895–0.949), indicating a significantly better performance compared to using 1,25(OH)_2_D alone (0.818 vs. 0.925, *p* < 0.001).

ROC analysis assessed the effectiveness of serum 1,25(OH)_2_D in identifying significant CAD (Figure 2). The AUC for 1,25(OH)_2_D in detecting significant CAD was 0.818 (95% CI: 0.777–0.855). The optimal threshold for 1,25(OH)_2_D was ≤16.6 pg/mL, with a sensitivity of 73.3% and a specificity of 83.5%. To construct the combined predictive model, a multivariable logistic regression analysis was performed including serum 1,25(OH)_2_D levels and other independent predictors of significant coronary artery disease identified in the multivariable analysis, namely age, current smoking status, diabetes, hypertension, and LDL-cholesterol. Predicted probabilities derived from this logistic regression model were then used to generate the ROC curve for the combined model. Notably, incorporating additional independent risk factors improved the predictive model, increasing the AUC to 0.925 (95% CI: 0.895–0.949), which significantly outperformed 1,25(OH)_2_D alone (0.818 vs. 0.925, *p* < 0.001).

## 4. Discussion

Conventional strategies for controlling cardiovascular risk factors, which have demonstrated efficacy in the general population, have shown only limited effectiveness in individuals with CKD [3]. Gaining a more comprehensive understanding of nontraditional contributors to cardiovascular disease, particularly those that emerge in the early stages of CKD, may facilitate the development of novel therapeutic interventions. Among these factors, vitamin D deficiency is commonly observed in CKD patients, as evidenced by decreased serum concentrations of 1,25(OH)_2_D and 25(OH)D. Notably, suboptimal vitamin D levels have been associated with an elevated risk of both all-cause and cardiovascular mortality in CKD, a trend that parallels observations in the general population [6,7]. Given this context, our study investigates the link between vitamin D levels and the occurrence and severity of CAD in CKD patients. In an analysis of 398 individuals undergoing CAG, we identified a significant and independent correlation between serum 1,25(OH)_2_D levels—and not 25(OH)D concentrations—and both the presence and extent of CAD. This association remained robust across various patient subgroups, independent of established CAD risk factors such as age, smoking, diabetes, hypertension, and LDL cholesterol levels. These findings build upon previous studies highlighting an independent association between vitamin D status and cardiovascular disease in CKD patients.

The key conclusion of our study is that lower serum 1,25(OH)_2_D levels independently correlate with both the presence and severity of CAD in CKD patients. Several previous studies have explored the connection between vitamin D deficiency and CAD in populations without CKD. Investigations by Akin et al., Liew et al., and Verdoia et al. have demonstrated an association between reduced serum 25(OH)D levels and the presence or extent of CAD in individuals undergoing CAG [8,9,14]. Similarly, Joergensen et al. reported a link between low 25(OH)D levels and asymptomatic CAD in high-risk type 2 diabetes patients [10], while Somuncu et al. found that 25(OH)D deficiency serves as an independent predictor of severe CAD in individuals with myocardial infarction [12]. Unlike these prior studies, which focused on 25(OH)D, our research specifically examined the association between 1,25(OH)_2_D and CAD, with a particular emphasis on CKD patients. To the best of our knowledge, this is the first study to explore the relationship between 1,25(OH)_2_D levels and CAD in this patient population.

Our study demonstrated an independent association between serum 1,25(OH)_2_D levels and both the presence and severity of CAD, even after adjusting for major traditional risk factors. This suggests that low serum 1,25(OH)_2_D levels may serve as nontraditional risk factors contributing directly to CAD development in CKD patients. However, the precise pathophysiological mechanisms underlying this association remain incompletely understood. Several potential mechanisms have been proposed to explain this relationship. Atherosclerosis, a key driver of CAD, is closely linked to inflammation, and vitamin D is thought to exert protective effects against CAD by modulating the inflammatory response [15,16,17]. Specifically, vitamin D reduces the expression of pro-inflammatory cytokines, leading to decreased CRP levels and limiting macrophage-derived foam cell formation, a critical step in atherosclerosis progression [16,17,18]. Beyond its anti-inflammatory actions, 1,25(OH)_2_D is thought to impact lipid metabolism by inhibiting LDL uptake in macrophages, achieved through the suppression of scavenger receptor expression on their surfaces [17]. In addition to atherosclerosis, vascular calcification is another frequent pathological change observed in atherosclerotic coronary artery stenosis, with almost all angiographically significant lesions exhibiting some level of calcification [8,19]. While our study did not directly measure coronary artery calcification, previous studies have suggested that vitamin D deficiency may increase the risk of developing coronary calcification [20]. Collectively, our results imply that 1,25(OH)_2_D deficiency in CKD patients may promote CAD progression by enhancing both atherosclerosis and vascular calcification. Further research is needed to clarify the precise mechanisms behind this association in CKD patients.

In patients with CKD, systemic inflammation and vascular calcification represent key, interrelated mechanisms contributing to accelerated atherosclerosis and coronary artery disease. CKD is characterized by a chronic pro-inflammatory state driven by uremic toxins, oxidative stress, and immune dysregulation, all of which promote endothelial dysfunction and plaque formation. In this context, reduced levels of 1,25(OH)_2_D may further exacerbate inflammation by increasing pro-inflammatory cytokine production and impairing macrophage and endothelial cell function. In addition, disturbances in mineral metabolism commonly observed in CKD, including phosphate retention and secondary hyperparathyroidism, contribute to vascular smooth muscle cell osteogenic transformation and vascular calcification. Although coronary artery calcification was not directly assessed in this study, the observed association between low 1,25(OH)_2_D levels and coronary artery disease severity may reflect, at least in part, the combined effects of enhanced inflammation and accelerated vascular calcification in CKD.

To our knowledge, this is the first study to demonstrate an independent association between serum 1,25(OH)_2_D levels and both the presence and severity of coronary artery disease specifically in patients with CKD. Unlike previous studies that primarily focused on 25(OH)D, our findings highlight the potential clinical relevance of assessing the biologically active form of vitamin D in this population. From a clinical perspective, serum 1,25(OH)_2_D may serve as a non-traditional biomarker for cardiovascular risk assessment in patients with CKD. Measurement of 1,25(OH)_2_D could potentially aid in identifying high-risk individuals who may benefit from closer cardiovascular surveillance or targeted vitamin D-related interventions. In addition, 1,25(OH)_2_D levels may be useful for monitoring the biological response to active vitamin D supplementation, although prospective studies are required to determine whether such strategies can improve cardiovascular outcomes. While reduced eGFR is a major cardiovascular risk factor in CKD, the independent association of 1,25(OH)_2_D with coronary artery disease after adjustment for kidney function suggests that this biomarker captures additional CKD-specific pathophysiological information beyond eGFR alone.

Several important considerations should be taken into account when interpreting the findings of this study. First, given the retrospective and cross-sectional design, the observed association between serum 1,25(OH)_2_D levels and coronary artery disease should be interpreted as associative rather than causal. It is possible that reduced 1,25(OH)_2_D levels reflect disease severity or systemic inflammation in patients with advanced CAD, raising the possibility of reverse causality. Clarifying the temporal relationship between vitamin D metabolism and CAD progression will require prospective longitudinal studies and randomized controlled trials. Second, the study population consisted of patients with CKD who underwent elective coronary angiography for evaluation of chest pain or suspected myocardial ischemia. Consequently, the cohort may be enriched for individuals with symptomatic or more advanced CAD, and the present findings may not be directly applicable to asymptomatic patients or the broader CKD population. Third, serum 1,25(OH)_2_D is closely linked to kidney function and parameters of CKD–mineral bone disorder, including phosphate and PTH. Although the association between 1,25(OH)_2_D levels and CAD remained significant after multivariable adjustment, residual confounding related to kidney disease severity and disordered mineral metabolism cannot be fully excluded. In this context, 1,25(OH)_2_D may represent not only an independent risk factor but also an integrated biomarker reflecting the overall burden of CKD-related metabolic and vascular disturbances. In addition, CAD severity was assessed using conventional coronary angiography and the Gensini score, which primarily reflect luminal stenosis. In patients with CAD, angiographic findings may underestimate the total atherosclerotic burden due to diffuse disease, heavy vascular calcification, and microvascular dysfunction, which are not adequately captured by luminal assessment alone. Finally, the ROC-derived cut-off value for serum 1,25(OH)_2_D was intended to demonstrate discriminatory performance within the study population and should be regarded as exploratory. Given potential inter-assay variability and laboratory-specific reference ranges, this threshold should not be interpreted as a basis for clinical decision-making without prospective validation.

Although this study identified a relationship between vitamin D deficiency and CAD in individuals with CKD, it remains uncertain whether vitamin D supplementation can influence the progression or onset of CAD in this population. Previous randomized controlled trials (RCTs) have largely failed to demonstrate a significant benefit of vitamin D therapy on CAD-related outcomes, including mortality [21]. However, some studies have suggested a possible protective effect of vitamin D in reducing CAD severity [21]. For instance, Wu et al. explored whether a six-month daily administration of 0.5 µg 1,25(OH)_2_D could improve CAD severity [22]. Their findings revealed a significant reduction in disease severity, as indicated by a notable decrease in SYNTAX scores (−3.9; *p* < 0.001). Importantly, this study was the only RCT that specifically evaluated the impact of 1,25(OH)_2_D administration, the active form of vitamin D, on CAD. Compared to cholecalciferol, this form of vitamin D is more biologically potent and may be better suited for correcting vitamin D insufficiency. Furthermore, our findings demonstrated that 1,25(OH)_2_D, rather than 25(OH)D, was significantly associated with CAD severity, raising the possibility that supplementation with 1,25(OH)_2_D might have a greater impact on disease progression. Further research is necessary to determine whether treatment with 1,25(OH)_2_D can positively affect CAD outcomes, including disease severity.

Several limitations of this study should be acknowledged. First, due to the retrospective design and inclusion of only patients who underwent elective coronary angiography, selection bias cannot be excluded, and the findings may not be generalizable to the broader CKD population. Second, vitamin D measurements were obtained as part of routine clinical practice rather than batch-analyzed samples, which may introduce some degree of assay variability. However, all assays were performed using standardized methods with consistent internal quality control. Third, quantitative coronary artery calcification data were not available; therefore, we were unable to directly evaluate the relationship between 1,25(OH)_2_D levels and coronary calcification, which may represent one of the proposed mechanistic pathways linking vitamin D deficiency to CAD. Finally, as this was a single-center study, the findings may not be fully generalizable to other populations or healthcare settings, and multicenter prospective studies are warranted to validate our results.

Nevertheless, this study offers several key strengths. First, instead of measuring 25(OH)D levels, we assessed serum 1,25(OH)_2_D concentrations. Since 1,25(OH)_2_D is the biologically active form of vitamin D that binds directly to the vitamin D receptor, it provides a more accurate representation of vitamin D’s physiological role, whereas 25(OH)D primarily serves as an indicator of vitamin D storage [5]. Therefore, our findings provide a more direct physiological insight compared to previous studies that focused on 25(OH)D. Second, the multivariable analysis incorporated adjustments for well-established risk factors of CAD, such as age, smoking, diabetes, hypertension and, LDL cholesterol, thereby reinforcing the independent association between 1,25(OH)_2_D and CAD in CKD patients. Third, beyond identifying this independent correlation, we also determined optimal threshold values for serum 1,25(OH)_2_D levels that may help predict significant CAD, suggesting its potential role as a biomarker in CKD patients.

## 5. Conclusions

In conclusion, this study demonstrates that serum 1,25(OH)_2_D levels are independently associated with both the presence and severity of CAD in patients with CKD. These findings suggest that 1,25(OH)_2_D may represent a clinically relevant, non-traditional biomarker for cardiovascular risk stratification in this high-risk population. Future prospective studies and randomized controlled trials are warranted to determine whether assessment and targeted modulation of 1,25(OH)_2_D levels can improve cardiovascular outcomes and inform personalized preventive strategies in patients with CKD.

## Figures and Tables

**Table 1:** Baseline characteristics of the study population according to tertiles of serum 1,25(OH)_2_D values (*n* = 398).

	Tertile 1 (<11.5 pg/mL) (*n* = 132)	Tertile 2 (11.5–19.7 pg/mL) (*n* = 133)	Tertile 3 (>19.7 pg/mL) (*n* = 133)	*p*
Age (years)	64.5 (58.0–73.0)	59.0 (54.0–67.0)	58.0 (50.0–66.0)	<0.001
Sex, male [*n* (%)]	62 (47.0%)	72 (54.1%)	67 (50.4%)	0.506
Current smoking [*n* (%)]	19 (14.4%)	23 (17.3%)	18 (13.5%)	0.668
Diabetes [*n* (%)]	97 (73.5%)	68 (51.1%)	48 (36.1%)	<0.001
Hypertension [*n* (%)]	112 (84.8%)	87 (65.4%)	70 (52.6%)	<0.001
Body mass index (kg/m^2^)	23.1 (21.8–25.2)	23.7 (21.6–25.4)	23.9 (22.3–25.8)	0.270
eGFR (ml/min/1.73 m^2^)	21.4 (13.2–30.6)	25.7 (15.6–36.4)	31.9 (24.0–43.4)	<0.001
Urinary albumin (g/g Cr)	2.31 (1.49–3.44)	2.17 (1.39–3.19)	1.76 (1.14–2.50)	<0.001
Albumin (g/dL)	3.9 (3.5–4.1)	3.9 (3.6–4.1)	3.8 (3.6–4.0)	0.499
Uric acid (mg/dL)	5.0 (4.2–6.2)	5.5 (4.4–6.9)	5.2 (4.1–6.1)	0.078
Calcium (mg/dL)	9.0 (8.8–9.3)	9.0 (8.7–9.3)	9.0 (8.8–9.6)	0.814
Phosphate (mg/dL)	3.8 (3.2–4.4)	3.6 (3.1–4.5)	3.4 (3.1–3.9)	0.001
LDL-cholesterol (mg/dL)	142 (112–166)	131 (103–157)	110 (86–144)	<0.001
Hemoglobin (g/dL)	10.9 (9.9–12.2)	11.6 (9.8–13.8)	11.9 (10.6–13.1)	<0.001
HsCRP (mg/dL)	0.7 (0.5–1.0)	0.7 (0.4–0.9)	0.5 (0.3–0.8)	0.003
Intact PTH (pg/mL)	109 (61–162)	81 (52–132)	70 (43–129)	<0.001
Significant CAD *	126 (95.5%)	93 (69.9%)	58 (43.6%)	<0.001
Gensini score	74 (42–120)	32 (10–72)	10 (6–20)	<0.001

* Significant CAD was defined as a stenosis of 50% or greater in at least one of the main coronary arteries. Data are presented as median (interquartile range) or (*n*, %). CAD: coronary artery disease; HsCRP, High sensitivity C-reactive protein.

**Table 2:** Univariable and multivariable analyses for variables associated with significant CAD * in the study participants (*n* = 398).

	Univariable		Multivariable	
	Odds Ratio (95% CI)	*p*	Odds Ratio (95% CI)	*p*
Age (1 year)	1.08 (1.05–1.10)	<0.001	1.05 (1.02–1.09)	0.003
Sex, male (vs. female)	0.91 (0.60–1.40)	0.680	1.58 (0.81–3.05)	0.175
Current smoking (vs. no smoking)	4.16 (1.93–8.98)	<0.001	7.36 (2.41–22.46)	<0.001
Diabetes (vs. no diabetes)	8.68 (5.19–14.50)	<0.001	3.85 (1.91–7.77)	<0.001
Hypertension (vs. no hypertension)	7.14 (4.44–11.48)	<0.001	3.52 (1.79–6.95)	<0.001
Body mass index (kg/m^2^)	0.94 (0.86–1.02)	0.939		
eGFR (1 mL/min/1.73 m^2^)	0.93 (0.92–0.95)	<0.001	0.96 (0.93–0.99)	0.032
Urinary albumin (1 g/g Cr)	1.58 (1.27–1.98)	<0.001	0.86 (0.61–1.22)	0.932
Albumin (1 g/dL)	0.77 (0.42–1.40)	0.390		
Calcium (1 mg/dL)	1.02 (0.61–1.71)	0.939		
Phosphate (1 mg/dL)	1.45 (1.11–1.88)	0.006	0.72 (0.47–1.11)	0.141
LDL-cholesterol (mg/dL)	1.03 (1.02–1.04)	<0.001	1.02 (1.01–1.03)	<0.001
Hemoglobin (1 g/dL)	0.88 (0.80–0.98)	0.015	1.01 (0.85–1.19)	0.955
HsCRP (1 mg/dL)	2.48 (1.43–4.30)	0.001	1.94 (0.86–4.39)	0.113
Intact PTH (10 pg/mL)	1.05 (1.01–1.08)	0.010	1.00 (0.94–1.07)	0.921
25(OH)D (ng/mL)	0.98 (0.95–1.01)	0.117		
1,25(OH)_2_D (pg/mL)	0.89 (0.87–0.91)	<0.001	0.89 (0.86–0.93)	<0.001

* Significant CAD was defined as a stenosis of 50% or greater in at least one of the main coronary arteries. CAD, coronary artery disease; eGFR, estimated glomerular filtration rate; HsCRP, High sensitivity C-reactive protein; LDL, low density lipoprotein; PTH, parathyroid hormone; 25(OH)D, 25-hydroxyvitamin D; 1,25(OH)_2_D, 1,25-dihydroxyvitamin D.

**Table 3:** Univariable and multivariable analyses for variables associated with Gensini score in the study participants (*n* = 398).

	Univariable		Multivariable	
	r *	*p*	β ^†^	*p*
Age (1 year)	0.408	<0.001	0.203	<0.001
Sex, male	−0.155	0.002	−0.063	0.077
Current smoking	0.179	<0.001	0.126	<0.001
Diabetes	0.479	<0.001	0.187	<0.001
Hypertension	0.413	<0.001	0.149	<0.001
Body mass index (kg/m^2^)	−0.048	0.344		
eGFR (ml/min/1.73 m^2^)	−0.401	<0.001	−0.037	0.465
Urinary albumin (g/g Cr)	0.210	<0.001	−0.044	0.290
Albumin (g/dL)	−0.063	0.208		
Calcium (mg/dL)	−0.036	0.469		
Phosphate (mg/dL)	0.207	<0.001	0.010	0.811
LDL-cholesterol (mg/dL)	0.437	<0.001	0.162	<0.001
Hemoglobin (g/dL)	−0.169	0.001	−0.051	0.197
HsCRP (mg/dL)	0.194	<0.001	0.016	0.673
Intact PTH (pg/mL)	0.218	<0.001	0.060	0.146
25(OH)D (ng/mL)	0.067	0.182		
1,25(OH)_2_D (pg/mL)	−0.539	<0.001	−0.329	<0.001

* r, Pearson’s correlation coefficients; ^†^ β, Standardized regression coefficient; eGFR, estimated glomerular filtration rate; HsCRP, High sensitivity C-reactive protein; LDL, low density lipoprotein; PTH, parathyroid hormone; 25(OH)D, 25-hydroxyvitamin D; 1,25(OH)_2_D, 1,25-dihydroxyvitamin D.

## Data Availability

The datasets used and/or analyzed during the current study are available from the corresponding author on reasonable request.
